# A Qualitative Study Documenting Black Birthing Individuals' Perspectives on the Disproportionate Rate of Preterm Birth in the Black Community

**DOI:** 10.1089/whr.2021.0116

**Published:** 2022-05-10

**Authors:** Sarahn M. Wheeler, Khaila Ramey-Collier, Kelley E.C. Massengale, Konyin Adewumi, Thelma A. Fitzgerald, Teresa Swezey, Geeta K. Swamy, Amy Corneli

**Affiliations:** ^1^ Division of Maternal and Fetal Medicine, Department of Obstetrics and Gynecology, Duke University School of Medicine, Durham, North Carolina, USA.; ^2^ Duke University School of Medicine, Durham, North Carolina, USA.; ^3^ Diaper Bank of North Carolina, Durham, North Carolina, USA.; ^4^ Department of Population Health Sciences, Duke University School of Medicine, Durham, North Carolina, USA.

**Keywords:** preterm birth, premature birth, race, disparities, inequity, qualitative

## Abstract

**Background:**

Compared with all other racial and ethnic groups, the rate of preterm birth (PTB) is 50% higher among non-Hispanic Blacks (NHB). There are limited published data focused on the etiology of the racial disparity in PTB from the perspective of Black birthing individuals who have had a lived experience with PTB.

**Methods:**

To gain insights into the etiology of the race disparity in PTB from the NHB patient's perspective, we conducted a qualitative descriptive study with NHBs who have a history of PTB. We conducted both focus group discussions (FGDs), in-depth interviews (IDIs), and used applied thematic analysis to analyze the data.

**Results:**

Seven individuals participated in 3 FGDs and 15 individuals participated in an IDI. The majority of participants named stress as a contributor to PTB among NHBs. Participants described that stress becomes an ongoing cycle with a cumulative effect on health. Three primary sources of stress were identified: (1) individual including stress from lack of personal wellness, (2) relational stress from intimate partner and familial relationships, and (3) community-level stress from occupations and societal expectations.

**Conclusion:**

Uncovering NHB patient's perspectives on the etiology of PTB is a critical step to develop interventions that mitigate the disparity impacting the Black community. Our findings suggest that multilevel interventions targeting individual-, relational-, and community-level stress may be necessary to reduce rates of PTB among NHB individuals.

## Introduction

Preterm birth (PTB, delivery before 37 weeks of gestation) is a leading cause of neonatal death.^1^ Babies that survive can face lifelong consequences ranging from respiratory infections, visual impairment, and learning delays.^1^ On average, each PTB costs $76,000 due to medical care for the infant, lost wages for caregivers, and special education needs during childhood.^2^ These costs translate to a conservative estimate of $26 billion per year in the United States due to PTB.^3^ The impact of PTB is devastating, yet the burden of prematurity is not distributed equally across the United States.

Compared with all other racial and ethnic groups, the rate of PTB is 50% higher among non-Hispanic Black (NHB) birthing individuals.^4^ In 2020, the U.S. PTB rate was 10.1% overall. Among individuals who identify as non-Hispanic White (NHW), the rate was 9.2%, whereas the rate was 14.0% among NHB birthing individuals.^5^ NHB babies born preterm are nearly four times more likely to die in the first 28 days of life compared with NHW babies.^4,6^

Patients with prior PTB have a twofold increased risk of PTB in a subsequent pregnancy.^1^ The risk of recurrent PTB is fourfold higher for NHBs than for NHWs.^7^ Scientists have explored numerous etiologies to explain the disproportionate rate of PTB in the NHB community. Etiologies ranging from progesterone receptor mutations,^8^ alterations in the cervical–vaginal microbiome^9^ and maternal behavioral patterns^10^ have all been explored, yet none of these theories completely explains the persistent disparity.

Race is a social construct that has a profound impact on lived experience at multiple levels. At the societal level, residential segregation^11^ and exposures to environmental toxins^12^ contribute to PTB. At the health care institution level, access to high-quality care is important; yet even in the military where patients have universal health care coverage, disparities persist.^13^

At the individual level, data suggest implicit biases affect patient–provider communication and health outcomes for Black patients^14,15^ even when access is equal. Racial disparities in PTB persist even when controlled for socioeconomic status and education.^16^ There is an increased understanding that lived experience with inequities on all levels of society, collectively termed racism,^11,17,18^ are the root cause of racial health disparities, including PTB.

Despite the growing evidence linking NHB's lived experiences and negative health outcomes,^11,19,20^ there are limited published data focused on important health disparities, such as PTB, from NHB patient's perspective. The Black Mommas Matter Alliance, a nationally renowned organization leading efforts for maternal health equity, advise listening to Black women as their top recommendation toward setting the standard of holistic care for Black birthing individuals.^21^ Interventions that redesign obstetric care centering Black patient's needs are imperative to mitigate the disparity in PTB. Therefore, we conducted a qualitative research study with NHB patients who have a history of PTB.

## Methods

### Study design

We conducted a qualitative descriptive study using focus group discussions (FGDs) and in-depth interviews (IDIs). We asked self-described NHB individuals to describe reasons why Black patients are more likely to deliver babies early compared with other races. We asked this question as part of a larger mixed methods study that was conducted to identify patient-perceived interventions to improve uptake and adherence to 17-hydroxyprogesterone caproate (17-P) for PTB prevention. The methods and results of the original study have previously been published.^22^ The study was approved by the Duke Health Institutional Review Board (Pro00084334).

We originally designed the study using FGDs only to capitalize on the group dynamic and brainstorm solutions to mitigate barriers. However, due to low enrollment and scheduling challenges, we discontinued the FGDs and switched to IDIs. At the beginning of the FGD or IDI, the facilitator/interviewer informed participants that Black birthing individuals experience higher rates of PTB compared with individuals of other racial and ethnic groups. The facilitator/interviewer also informed participants that scientists are unsure exactly why Black individuals have higher rates of PTB compared with individuals of other races. Participants were encouraged to share their perspectives on why NHB birthing individuals are more likely to deliver preterm.

### Eligibility criteria and recruitment

Participants were eligible to participate in the FGDs or IDIs if they: (1) self-identified as NHB, (2) had a history of prior spontaneous PTB of a singleton gestation beyond 20 weeks, and (3) were currently or recently (within the past 2 years) pregnant with a singleton nonanomalous fetus beyond 20 weeks of gestation. Participants were only eligible to participate in one data collection activity (*i.e.*, either a FGD or an IDI).

Participants meeting the eligibility criteria were recruited during routine high-risk obstetrical clinic visits. FGDs were offered at a morning, afternoon, and evening time and a meal was provided. IDIs were offered at any time that was convenient to the participant when a trained interviewer was available. We were able to offer “on-demand” interviewing on the date of consent for participants who elected for immediate participation. Participants in both the FGD and IDI were compensated $20 for their time.

### Data collection

A description of our data collection procedures has previously been described.^22^ Briefly, demographic, obstetric, and general medical history information were collected from the patient's medical record. The FGDs were audiorecorded, facilitated by a trained moderator, attended by a note taker, and transcribed verbatim. The IDIs were also conducted by a trained interviewer, audiorecorded, and transcribed verbatim. In both the FGD and the IDIs, the facilitator described the unclear etiology of the disparity in PTB rates, then participants were asked for their thoughts on the etiology of the disparity. Participants were probed to describe their perspectives in more detail as appropriate.

### Analysis

Participant demographic characteristics were described using descriptive statistics. We used applied thematic analysis to analyze the qualitative data.^23^ As part of the larger study, we developed a codebook of structural and emergent codes after reviewing transcripts from the FGDs and IDIs. Structural codes were first applied to the transcripts to organize participant responses based on the FGD/IDI guide questions. Steps to apply codes and ensure intercoder reliability are described elsewhere.^22,24^

The current analysis focuses on data gathered within a single structural code—perceptions of PTB etiology. The structural code was then expanded to include additional and emergent codes after reading the IDI transcripts and applied these emergent codes to both the IDIs and FGDs as appropriate.^25^ We present the most salient themes that emerged during analysis. We analyzed FGD and IDI data separately. We report frequencies using the FGD as the unit of analysis (rather than reporting individual participants within the FGD) and IDIs are counted individually.

## Results

### Study population

The FGDs and IDIs were conducted between August 2017 and April 2018. Seven individuals meeting the inclusion criteria participated in the 3 FGDs (*n* = 3, 2, and 2, respectively) and 15 individuals participated in an IDI. All of the participants self-identified as women. The mean age of the participants was similar for the FGDs (32.6 years, standard deviation [SD] 5.7) and IDIs (31 years, SD 5.9).

The majority of participants were single, including 85% (*n* = 6) of the FGD participants and 67% (*n* = 10) of the IDI participants; the remaining FGD and IDI participants were married. All (*n* = 7) of the FGD participants had Medicaid/Medicare insurance, whereas 53% (*n* = 8) of the IDI participants had Medicaid/Medicare insurance and 47% (*n* = 7) had private insurance. Both the FGD and IDI participants had a median of one prior PTB. Five out of the seven FGD participants were pregnant at the time of their participation with a mean gestational age of 22.7 weeks. Thirteen of the 15 IDI participants were pregnant at the time of their participation with a mean gestational age of 28.3 weeks.

### Perceived etiology of PTB

When we analyzed themes from participant's perspectives on why NHBs experience more PTB, the majority of participants (*n* = 13 IDI, *n* = 3 FGD) named stress as a contributor. No other factor was named by >13 participants. One IDI participant commented, “*I feel as a Black female, not necessarily saying, you know, White people don't have stress while pregnant, but I believe we probably have a little more stress.*”

We identified three primary sources of stress—at the (1) individual, (2) relational, and (3) community levels. We describe them below, in order of most to least commonly cited:

### Individual

The most frequently reported source of individual-level stress was overall wellness, including nutrition (*n* = 10 IDI, *n* = 3 FGD) and exercise (*n* = 5 IDI, *n* = 2 FGD). This was followed by stress surrounding lack of preparation for a family (*n* = 4 IDI, *n* = 2 FGD) and age-related stress (*n* = 5 IDI, *n* = 1 FGD).

#### Wellness

Participants most often referred to their personal health and wellness as a possible etiology for increased PTB among Black birthing individuals. This was discussed in the context of nutrition and exercise with some participants mentioning the increased incidence of metabolic disease among NHB people. One FGD participant stated, “*I mean it probably is stress, how we handle stress, how we eat, the way we exercise. Just our daily lives are sometimes different than other ethnicities…It could be the same reason why African Americans are more prone to diabetes, high blood pressure…it's things that we eat; the lifestyle that we live…the amount of exercise that we have…I would think that would fall kind of in the same category.*”

Participants also noted lifestyle as an important component of wellness. This encompassed not having appropriate stress management skills, such as knowing when to remove oneself from a negative environment or avoiding overexertion during pregnancy. It also included self-care through regular health maintenance. An IDI participant reflected on the importance of, “*making sure we go to those physicals every year and just taking care of our bodies and ourselves. Some people tend to only go to the doctor if something's wrong, not for normal maintenance and maybe that has something to do with it as well.*” With respect to health care, participants noted that establishing a trusting relationship with a provider is a necessity and that physicians can provide preventive counseling and resources in addition to therapeutic treatment.

#### Preparation

Participants highlighted stress from lack of preparation for pregnancy. Preparation included being mentally prepared with realistic expectations about physical changes during pregnancy. For example, a FGD participant commented, “*I know when I was pregnant…I had pain and I was like I'm okay, this is a part of pregnancy? It's like you really don't know. A lot of people don't know what to expect.*” Participants also indicated that preparation from experiences during prior pregnancies left them better informed for future pregnancy*. An IDI participant commented,* “*You know I still have a little stress, but I didn't have the stress…[that] I did in my first pregnancy because you know I [was]…more prepared and you know I have a roof over my head, and I have a job,*”

#### Age

Participants (*n* = 5) made associations between youth and inexperience with managing stress during pregnancy. One FGD participant commented, “*…Black women might tend to have babies earlier when they are not at a place where they can handle as much stress, and that may play into a higher rate of preterm birth.*” Participants also discussed that with increasing age comes acquired wisdom from previous experiences leading to an understanding of the harmful effects of worrying and stress. Therefore, age was often described as a protective factor against stress during pregnancy. A FGD participant recounted, “*There are things that would set you off when you're younger and the things that you would just send your blood pressure through the roof; they no longer matter.*”

### Relational

The most frequently reported sources of relational stress, or stress associated with interactions with other individuals, were intimate partner relationships (*n* = 11 IDI, *n* = 5 FGD) followed by stress from familial relationships (*n* = 4 IDI, *n* = 3 FGD).

#### Intimate partner relationship

Participants referred to this type of stress as a function of limited support from their partner. Participants described that limited partner support leads to increased responsibilities and stress for pregnant Black people. An IDI participant claimed, “*To be honest with you, it's nothing that could really help prevent it until you get rid of the major stressors that's there. Until you know us as Black women train up our men or our young boys to be men in the household and stop expecting the woman to do it all you know, that's where it would stop.*”

Participants also remarked on how the need for support can influence decisions about remaining in a negative relationship. Another IDI participant commented, “*You see more single parent Black women than you see any other race and it's like I feel like that can kind of contribute because…you don't have enough support systems. I [see] a lot of young Black moms who you know they go through spouse abuse just to have somebody that say they love them and you know just to have somebody to you know help them out and stuff….*”

Participants also reflected on the lack of intimate partner involvement as a source of stigma leading to stress. For example, one FGD participant said, “*you have so many women who have children on their own, who go through an entire pregnancy on their own and I mean and then to deal with society, we're looked at a certain way because you're beneath people.*”

#### Familial

Participants noted that decreased partner support may require increased support from family and friends. As a result, one participant felt stress from transactional relationships with family. The IDI participant said, “*Basically I had to fend for myself…you know asking family members and stuff and then it becomes a headache because then…when my family members ask me to do something for them…I don't want to tell them no because…they pick my son up from school…I tell them no then I'm going to have to worry about how my son going to get out of school.*”

Additionally, participants (*n* = 9 IDI, *n* = 3 FGD) described decreased stress with a positive home environment as well as healthy responsibility sharing with their partner. One IDI participant said, “*when you got somebody to take half of that load it's not that much stress on you.*” Participants also discussed that support need not solely come from their partner, as healthy relationships with family and friends can be just as beneficial. Another IDI participant said, “*It doesn't have to be the daddy. Just the slightest bit of support carries you a long way.*”

Familial stress was also identified as stress from NHB female roles as caregivers within their family and unspoken rules surrounding the matriarchal role in NHB families. An IDI participant explained, “*a lot of African American women typically are the matriarchs in their family and so even if there is that support there, they're typically the head of the household and so most of the responsibility falls on them and even if you have a pending pregnancy, that responsibility is not likely to waiver or decrease.*” Participants described that a matriarchal family structure places increased responsibility on NHB women to be accountable for other family members and maintain stability for the family unit.

Furthermore, participants saw the pressure to lead the household to be uniquely burdensome for Black women. A FGD participant said, “*That was my situation and also having to do everything in the household you know, take care of everything and that's stressful. A lot of Black women, they don't have some of the luxuries that other races have when it comes to being grown up. You run everything so.*”

Participants' narratives also described that responsibility is regarded as a type of birthright and expectation. An IDI participant commented, “*In the Black community and the Black culture the main person that keeps the family together is always a Black female so that is a big stressor as far as finances, as far as getting the house together, as far as you know anything else and then you add any more to it like oh now I'm pregnant. Who suffers? The baby suffers.*”

### Community

The most commonly reported source of community stress involved occupational stress (*n* = 8 IDI, *n* = 1 FGD) followed by societal expectations (*n* = 4 IDI, *n* = 2 FGD).

#### Occupational

Participants recounted work-related experiences that caused increased stress during their pregnancy. This included, for example, supervisors' poor flexibility and lack of accommodations in scheduling more breaks or decreasing time standing. Some participants (*n* = 3) also experienced difficulties with getting time off to attend prenatal appointments.

One IDI participant stated, “*My decision was to give up my job for the health of my child…because I felt like any job that have to make you choose over your child, the health of your child and you going to a doctor's appointment that'll probably be an hour or two out of the day probably once a month is not really worth having.*” In making the decision between work and the health of both themselves and their child, participants also felt an added financial stress.

One IDI participant recalled that her boss said, “*you know you might want to quit your job and everything cause…in the beginning…I was so ill and sick…okay I'm ready to quit because, but then…I know that I can't quit because I have children at home to take care of you know I can't just afford to miss that time. I mean everything goes back to, I feel like everything goes back to stress.*” Thus, some women (*n* = 2) pushed through their physical limitations and continued to work due to financial strain. Another IDI participant commented, “*I can say it has something to do with, you know, probably stress financially … It's a lot, so I think that contributes to the facts of some women, you know, giving birth a little early.*”

#### Social expectations

Participants noted that NHB women are critical members within their households and the Black community and that the societal expectations of those gender roles can cause stress. As such, participants reflected on the expectation to be strong and steadfast in the face of adversity and loss. Subsequently, participants (*n* = 2) expressed that there is little time to reflect or grieve about struggles when others are relying on their strength and provisions.

An IDI participant explained, “*A lot of stressors that the Black community see is really not topic of conversation amongst us…It's like you know it's a stressor…It's like this is just what we go through as African American women…I hate to say it but a lot of our older generations put a lot of pressure onto the younger female…to be the head of the house instead of the man being the head of the house…You know just like we were built to be strong.*” Participants communicated that stress is common and expected, but is not frequently discussed within their communities. This also applied to processing pregnancy loss.

A FGD participant reflected, “*I know when I lost my son…my grandma…my great aunt…they came out and they were like well you know I lost a baby. And I never knew that… But you know, not knowing. Like I really felt like I was like alone…it's like a taboo to talk about it and I don't know why and I think that also plays a factor you know as to why it keeps happening because they don't want to talk, people aren't talking about it.*” In this discussion on restricted communication about stress and hardships, a FGD participant remarked on the expectation to endure and move on stating, “*You supposed to you know, go through something and whatever don't kill you make you stronger.*”

Finally, in addition to highlighting stress and the specific sources of stress, participants also reported that the stress becomes an ongoing cycle with a cumulative effect. An IDI participant explained, “*It tends to like be a downward spiral of just I guess bad things that go on*.”

## Comment

We asked NHB patients who have lived experience with PTB why they believe NHB patients experience more PTB. Stress was the predominant response. Participants' narratives on stress revealed that stress occurs at multiple levels—at the individual, relational, and community levels. Individual-level stressors, including wellness, pregnancy preparation, and early age at conception; relational included intimate partner relationships, and lack of male involvement in pregnancy, childrearing, and the household was an important underlying theme of relational; and occupation and societal expectations were community-level stressors described.

Stress as a potential etiology for the disproportionate PTB rate in NHBs is biologically plausible.^26^ There are biological changes in response to psychosocial stress, including increased activation of the maternal hypothalamic–pituitary–adrenal axis leading to a cascade of hormonal changes and increased corticotropin-releasing hormone (CRH).^27^ CRH increases prostaglandin production and prostaglandins initiate uterine contractions.^27,28^ In addition to biological plausibility, NHBs are disproportionately impacted by psychosocial stress during pregnancy. In a large cohort study of over 33,000 pregnancies from 19 states, Black birthing individuals were more likely to report stress overall and 163% more likely to report partner-related stressors.^29^

Further literature suggests cumulative life stressors, termed “*weathering*,” which are additive and resulting in poorer health.^30,31^ Although stress is more prevalent in NHBs, investigations examining the impact of psychosocial stress on PTB are mixed and some investigators have concluded that the impact of stress on PTB is modest.^32^ For example, the large cohort study that documented increased stressors in NHBs only demonstrated a modest link between the most severe traumatic stress, race, and PTB.^29^

The heterogeneity of findings linking race, stress, and PTB may be due, in part, to the variety of measurement instruments utilized when evaluating the relationship between psychosocial stress and PTB. In a systematic review of 138 studies measuring psychosocial stress and PTB, 85 different instruments were used to measure psychosocial stress.^32^

Measurement tools included different domains of stress ranging from anxious character traits, limited tangible or emotional supports, and exposure to specific stressful life events. Most studies evaluating stress and PTB rely on a single measure with a narrow definition of stress. Although our participants consistently pointed to stress as an etiology of PTB, sources of stress varied widely suggesting that studies evaluating one narrow aspect of stress may not have the sensitivity necessary to demonstrate the link between stress and PTB.

In addition to limited studies demonstrating a link between psychosocial stress and PTB, early attempts to increase social support *via* professional (social worker, nurse, midwife) or trained lay community workers did not reduce PTB.^33^ Although earlier attempts to increase social support had limited success and shed doubt on the link between stress and PTB, more recent interventions, including doulas and group prenatal care (GPNC) are promising. GPNC replaces traditional one-on-one provider visits with a series of structured group meetings led by a medically trained facilitator.^34^ The structured curriculum covers medical topics often addressed in traditional care such as weight gain, breastfeeding, and labor symptoms.

The GPNC curriculum also covers topics outside of the scope of traditional prenatal care, including many of the stressors identified in the current study. For example, the GPNC curriculum includes in-depth discussions about self-care, intimate partner and family relationships, and building support systems.^35^ The unique elements of GPNC have proven efficacious. In a randomized trial comparing traditional one-on-one prenatal visits with GPNC, the participants randomized to GPNC had a 33% lower PTB rate.^34^

Among the subset of African American participants, the PTB rate was reduced by over 40%.^34^ Similarly, doula support also targets many of the sources of stress that we identified in our analysis. Doulas are trained birth support workers who provide one-on-one support during pregnancy, labor, and postpartum.^36^ Doula training includes strategies to provide physical, emotional, partner, and family supports.^36^ In a large study, including over 65,000 births from administrative data sets, PTB was reduced by 22% among publicly insured patients with doula care.^37^

Our findings, in conjunction with the large body of research on PTB among NHBs and the potential role of stress in contributing to the higher rate, point to urgently needed policy-level change. In the current analysis and our previous work,^22,24^ NHBs consistently point to employment and structural barriers that contribute to disparities in PTB. Participants described choosing between their employment and their health during pregnancy. While there are some limited federal and varied state-level protections against pregnancy-related employment discrimination, benefits, including insurance and paid leave are variable. Our findings add to a growing conversation about dramatic policy-level changes that are needed to achieve equitable health outcomes in the United States.^38,39^

While we present novel data that document rarely reported perspectives from NHB birthing individuals who have experience with PTB, our findings must be considered in light of some important limitations. Since we collected data, the COVID 19 pandemic and the national conversation surrounding race and racism sparked by George Floyd's murder have had a profound impact on public perceptions about race and racism. It is unclear if the evolving national discourse would have influenced participant's responses.

Our findings may be limited by the relatively small sample size in the FGDs leading to an alternate approach with IDIs. However, our analysis revealed similar themes with the two different data collection approaches. Ultimately, we present rich descriptions of the potential etiologies for the disproportionate rate of PTB from the perspective of NHB patients personally impacted by PTB, which is lacking in the literature.

## Conclusion

Uncovering NHB birthing individual's perspectives surrounding the etiology of the racial disparity in PTB is a critical step to go beyond describing the disparity to developing targeted interventions to eliminate the disparity. Our participants made a strong statement about what they need for their health and the health of their families—that is, support. Our findings suggest that interventions designed to alleviate psychosocial stress may be uniquely impactful to address the persistent PTB disparity affecting the Black community. Further studies focused on interventions incorporating proven strategies, such as GPNC and doulas to address the social support needs identified by Black birthing individuals, are urgently needed.

## Abbreviations Used

17-P: 17-hydroxyprogesterone caproate
CRH: corticotropin-releasing hormone
FGDs: focus group discussions
GPNC: group prenatal care
HPA: hypothalamic–pituitary–adrenal
IDIs: in-depth interviews
NHB: non-Hispanic Blacks
NHW: non-Hispanic White
PTB: preterm birth
SD: standard deviation
